# Step length determines minimum toe clearance in older adults and people with Parkinson’s disease

**DOI:** 10.1016/j.jbiomech.2017.12.002

**Published:** 2018-04-11

**Authors:** Lisa Alcock, Brook Galna, Ruth Perkins, Sue Lord, Lynn Rochester

**Affiliations:** a Institute of Neuroscience, Newcastle University, Newcastle upon Tyne, United Kingdom; b Newcastle University Institute for Ageing, Newcastle University, Newcastle upon Tyne, United Kingdom; c School of Biomedical Sciences, Newcastle University, Newcastle upon Tyne, United Kingdom; d The Newcastle upon Tyne NHS Foundation Trust, Newcastle upon Tyne, United Kingdom

**Keywords:** Gait, Preferred and fast velocity, Minimum toe clearance, Ageing, Parkinson's disease, Falls, PD, Parkinson’s disease, MTC, minimum toe clearance, UPDRSIII, the Unified Parkinson’s Disease Rating Scale – Part 3, MOCA, Montreal Cognitive Assessment, H&Y, Hoehn and Yahr disease stage

## Abstract

Reduced foot clearance when walking may increase the risk of trips and falls in people with Parkinson’s disease (PD). Changes in foot clearance in people with PD are likely to be associated with temporal-spatial characteristics of gait such as walking slowly which evokes alterations in the temporal-spatial control of stepping patterns. Enhancing our understanding of the temporal-spatial determinants of foot clearance may inform the design of falls prevention therapies.

Thirty-six people with PD and 38 age-matched controls completed four intermittent walks under two conditions: self-selected and fast gait velocity. Temporal-spatial characteristics of gait and foot (heel and toe) clearance outcomes were obtained using an instrumented walkway and 3D motion capture, respectively. A general linear model was used to quantify the effect of PD and gait velocity on gait and foot clearance. Regression evaluated the temporal and spatial gait predictors of minimum toe clearance (MTC).

PD walked slower regardless of condition (p = .016) and tended to increase their step length to achieve a faster gait velocity. Step length and the walk ratio consistently explained the greatest proportion of variance in MTC (>28% and >33%, respectively) regardless of group or walking condition (p < .001).

Our results suggest step length is the primary determinant of MTC regardless of pathology. Interventions that focus on increasing step length may help to reduce the risk of trips and falls during gait, however, clinical trials are required for robust evaluation.

## 1 Introduction

Discrete modifications to step length and step time (or rate of steps; cadence) allow us to maintain an appropriate velocity for a given environment. Both the temporal and spatial control of gait is altered in people with Parkinson’s disease (PD) beyond that of normal ageing. People with PD walk more slowly, with a shorter step length and slower rate of steps, compared to age-matched controls (Morris et al., 1996, Carpinella et al., 2007, Galna et al., 2015). PD affects 1% of adults aged ≥60 years (de Lau and Breteler, 2006) and gait alterations are common (Morris et al., 1996, Hass et al., 2012, Galna et al., 2015) due to a range of motor impairments, including a reduced magnitude (hypokinesia) and speed (bradykinesia) of movement, axial rigidity and postural instability (Bloem et al., 2004, Morris et al., 2008, Rahman et al., 2008). People with PD often present with a shuffling gait (short, low steps) and consequently falls are common (Ashburn et al., 2001, Wood et al., 2002) with the majority of falls resulting from a trip (Gazibara et al., 2014).

The minimum toe clearance (mid-swing; MTC) has been considered an event linked to falls in older adults (Begg et al., 2007, Barrett et al., 2010) as the risk of making unanticipated contact with an environmental object/ground is high. A reduced foot clearance (and inadequate limb elevation) and its link with falls risk was first documented by James Parkinson in his seminal work characterising the motor symptoms of PD. He commented that “the legs are not raised to that height [when walking], or with that promptitude which the will directs, so that the utmost care is necessary to prevent frequent falls” (Parkinson, 1917). Fall risk may be further elevated, particularly for people with PD, due to compromised postural responses (Schoneburg et al., 2013, Park et al., 2015). Our recent work indicates that whilst MTC is not significantly affected in the early stages of PD, a lower MTC is associated with a reduced gait velocity and a shorter step in older adults with and without PD (Alcock et al., 2016). Gait alterations in people with PD are progressive and greater deficits in MTC may be observed when disease symptoms and motor impairments (i.e. reduced gait velocity and step length) are more prominent. In support, increasing set velocities using a motorised treadmill resulted in a higher MTC in people with moderately advanced PD (Cho et al., 2010). Considering the constant velocity that walking on a motorised treadmill imposes, it is important to determine whether MTC is elevated during overground walking when increasing walking speed.

The aims of this study were to (1) quantify the alterations in foot clearance when instructed to walk quickly; and (2) evaluate whether MTC is primarily determined by step length, step velocity or step time in people with PD. We hypothesised that people with PD would demonstrate an elevated MTC when asked to walk faster, and that an increased MTC would be most strongly associated with longer steps rather than changes in temporal characteristics.

## 2 Methods

### 2.1 Participants

Thirty-six people with PD ($\overset{¯}{\text{x}}$ [SD]age 70.1[9.7]years, height 1.71[0.08]m and mass 78.2[16.7]kg, 26[72%] males) and 38 controls of similar age and sex ($\overset{¯}{\text{x}}$ [SD]age 72.4[7.8]years, height 1.71[0.09]m and mass 80.9[13.4]kg, 21[55%] males) underwent clinical gait analysis as part of the ICICLE-GAIT study (Rochester et al., 2014, Galna et al., 2015). ICICLE-GAIT is a collaborative study with ICICLE-PD, an incident cohort study (Incidence of Cognitive Impairment in Cohorts with Longitudinal Evaluation–Parkinson’s disease) which recruited participants between June 2009 and December 2011 and followed them longitudinally every 18-months (Khoo et al., 2013). Ethical approval was granted from the local NHS Research Ethics Committee (Ref: 09/H0906/82) and all participants provided written informed consent. PD participants were recruited from the local Movement Disorders Clinic and community resources were used to recruit controls that had no significant cognitive or movement impairment. People with PD were excluded if they: presented with parkinsonism syndromes other than PD (i.e. dementia with Lewy bodies, vascular or drug-induced parkinsonism); had multiple system atrophy; progressive supranuclear palsy; or were unable to communicate sufficiently in English. Data presented in this study represent a cross-sectional analysis of 54 and 72-month assessments. People with PD were assessed whilst optimally medicated approximately 1-hour post dopaminergic medications. PD severity was quantified using the Hoehn and Yahr stage (H&Y) (Hoehn and Yahr, 1967) and motor function was evaluated using Part III of the Movement Disorder Society Unified Parkinson’s Disease Rating Scale (UPDRS III) (Goetz et al., 2008). Global cognitive function was quantified using the Montreal Cognitive Assessment (MOCA) due to its increased sensitivity with people with PD (Zadikoff et al., 2008).

### 2.2 Protocol

Participants attended the Gait Laboratory wearing comfortable, flat soled shoes and performed four 10-metre straight walks at a preferred velocity and then four walks at a fast velocity. Trial order was standardised to avoid the potential carryover effect of fast walking velocity onto their preferred walking velocity, similar to protocols used in previous research (Hirasaki et al., 1999, Chui and Lusardi, 2010). Temporal-spatial gait characteristics were obtained from a 7-m instrumented walkway (GAITRite®, Platinum model v.4.5, USA, 240 Hz) which was placed centrally within the walkway to ensure the collection of steady-state gait (2-metres was covered before and after the mat to allow for acceleration and deceleration). To measure foot clearance, 14 mm reflective markers were affixed on the shoe above the dorsum of the second hallux (toe) and the calcaneus (heel). Trajectories were recorded using a 14 camera three-dimensional motion capture system sampling at 100 Hz (Bonita 10 cameras, VICON© Nexus software v.2.2.3, Oxford, UK).

### 2.3 Data analysis

Procedures for data extraction of temporal-spatial and foot clearance outcomes have been previously described (Alcock et al., 2016). Briefly, temporal-spatial gait characteristics (gait velocity, swing velocity, step time, swing time and step length) were obtained from footfall data and extracted using Microsoft® Access 2007. A walk ratio (step length[m]/cadence[steps/min] (Sekiya and Nagasaki, 1998)) was calculated for each participant and condition as a velocity-independent index of gait control (Rota et al., 2011). Marker trajectories were identified and smoothed (Woltring filter, Mean square error 20 mm, Vicon© Nexus v.2.2.3). Foot clearance outcomes were extracted using a custom-built algorithm implemented in MATLAB® (R2015a, Mathworks, Natick, MA) (Alcock et al., 2016). Vertical displacement of the heel and toe were segmented into gait cycles using a vertical velocity threshold of the heel trajectory of 250 mm/s to identify heel strike. An offset correction was applied to account for variation in marker placement and to ensure that when the foot was flat on the floor (i.e. during mid-stance), foot clearance was 0 mm. A vertical offset corrected the heel marker and an angular offset aligned the toe marker with the heel. The following outcomes were extracted: the maximum vertical toe displacement during the first and second half of swing; the minimum vertical toe displacement mid-swing (MTC); and the peak vertical heel displacement. Peaks were identified when a data point was larger than the three samples before and after to avoid erroneous peak detection. All extracted gait cycles where visually checked prior to further analyses. The take-off (toe) and landing (heel) gradients were also calculated and defined as the change in vertical displacement, divided by the change in time (5% of the start or end of the swing phase, respectively). Variability in foot clearance was not calculated due to an insufficient number of steps collected across the four intermittent walks (a minimum of 200 steps have been recommended to be sufficient for calculating variability (Hamacher et al., 2014)).

### 2.4 Statistical analysis

A normal distribution was generally confirmed for all group mean data through visual inspection of the histograms and evaluating skewness. A General Linear Model was used to identify the influence of group (Control *vs*. PD) and condition (preferred *vs*. fast velocity) on temporal-spatial gait and foot clearance (Aim 1). No significant differences in age, height, mass, sex or global cognition were observed between groups and thus were not controlled for in the model. Change in gait and foot clearance between conditions were also calculated in relative (%) (i.e. step length_Fast_ − step length_Preferred_/step length_Fast_ ∗ 100) terms. Linear regression was used to quantify the variance in MTC explained by temporal-spatial gait characteristics. Two outliers were identified (one control and one participant with PD) as cases with standardised residuals of >3SD but they did not considerably influence the model (explained variance or beta coefficient) and remain included in the analysis. Correlations between temporal-spatial gait and foot clearance are presented in the Supplementary Material.

## 3 Results

No significant group differences in global cognition (MOCA) were observed (control group = 26.6[3.3], PD group = 25.7[3.6]). The PD group presented with mild-to-moderate disease severity, with 89% (n = 32) of the sample rated as Hoehn and Yahr stage II and a group mean UPDRS III score of 36.9[12.6]. A similar number of steps (mean[SD]) were collected for both groups when walking at a preferred (Control group: 29[8]steps, PD group: 31[9]steps) and a fast (Control group: 25[6]steps, PD group: 27[8]steps) velocity.

### 3.1 Alterations in temporal spatial gait and foot clearance during fast walking

**Fig. 1:**
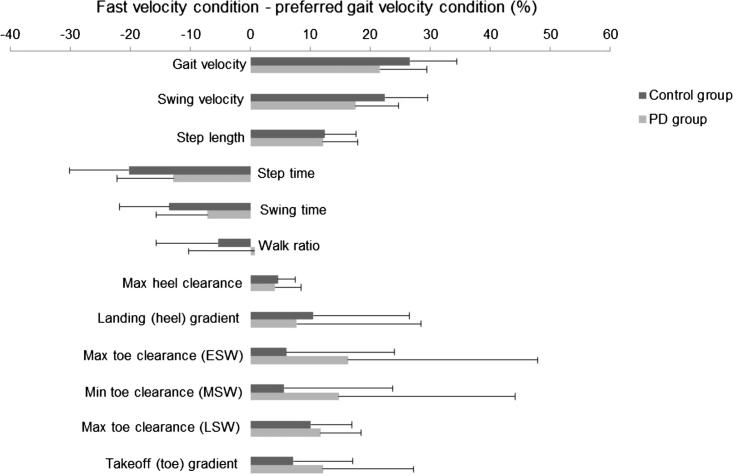
Relative difference (%) in temporal-spatial components of gait and foot clearance parameters with error bars indicating group standard deviation. ESW, MSW and LSW denote early, mid- and late swing, respectively.

Evaluation of within-group (condition) and between-group (control *vs*. PD) differences in temporal-spatial gait characteristics and foot clearance are presented in Table 1. Barring the walk ratio, condition had a main effect on all gait and foot clearance outcomes (Fig. 1), the majority of which significantly increased with faster velocities (p < .001). In contrast, step time reduced with increasing velocity (p < .001). Group differences indicated that people with PD walked more slowly, with a slower swing velocity and reduced peak toe clearance (late swing) regardless of condition. Interestingly, people with PD walked with a reduced (shorter) swing time compared to controls at their preferred velocity and with an increased (longer) swing time compared to controls at their fast velocity (p = .002). An interaction was observed for the walk ratio highlighting that people with PD increased their step length relative to their cadence from preferred to fast gait more so than controls (p = .018). Interactions were also observed for step time, swing time, gait velocity and swing velocity, revealing a greater group difference during the fast walking condition.

**Table 1:** Statistical differences in temporal-spatial characteristics of gait and foot clearance outcomes due to group and walking condition.

	Controls (n = 38)			PD (n = 36)			General linear model		
	Preferred velocity	Fast velocity	Difference (Fast – Preferred)	Preferred velocity	Fast velocity	Difference (Fast – Preferred)	Condition	Group	Condition × Group
Gait velocity (m/s)	1.28 [0.20]	1.76 [0.33]	0.48 [0.21]	1.20 [0.26]	1.53 [0.33]	0.34 [0.15]	*<.001*	*.016*	*.001*
Swing velocity (m/s)	1.76 [0.23]	2.28 [0.36]	0.52 [0.22]	1.67 [0.27]	2.03 [0.33]	0.36 [0.16]	*<.001*	*.014*	*.001*
Step length (m)	0.68 [0.10]	0.79 [0.13]	0.10 [0.05]	0.64 [0.18]	0.73 [0.13]	0.09 [0.05]	*<.001*	.075	.335
Step time (msec)	539.0 [47.9]	450.8 [52.4]	−88.2 [35.9]	540.9 [51.8]	482.4 [59.0]	−58.5 [44.7]	*<.001*	.145	*.002*
Swing time (msec)	391.4 [33.9]	346.5 [39.1]	−44.9 [23.5]	384.7 [40.3]	361.0 [45.0]	−23.7 [33.1]	*<.001*	.646	*.002*
Walk ratio (Length/Cadence)	0.00615 [0.00102]	0.00591 [0.00121]	−0.0002 [0.0005]	0.00577 [0.00113]	0.00588 [0.00136]	0.0001 [0.0008]	.373	.438	*.018*
Total number of steps	1096	956	−140	1113	975	−138	*<.001*	.226	.909
Peak heel clearance (mm)	253.8 [31.5]	266.0 [33.1]	12.2 [7.7]	253.4 [29.1]	264.1 [26.8]	10.7 [11.1]	*<.001*	.870	.489
Landing (heel) gradient	2.34 [0.68]	2.75 [1.09]	0.41 [0.58]	2.14 [0.69]	2.39 [0.77]	0.25 [0.58]	*<.001*	.130	.249
Max toe clearance (ESW; mm)	28.9 [6.9]	31.6 [8.6]	2.65 [5.48]	30.3 [8.9]	34.8 [10.9]	4.04 [6.67]	*<.001*	.296	.331
Min toe clearance (MSW; mm)	28.1 [7.1]	30.5 [8.3]	2.34 [5.20]	29.6 [9.0]	33.7 [10.8]	3.55 [6.20]	*<.001*	.285	.370
Max toe clearance (LSW; mm)	139.0 [29.4]	155.8 [36.1]	16.75 [13.37]	119.4 [30.6]	135.7 [35.1]	16.27 [10.60]	*<.001*	*.010*	.864
Take-off (toe) gradient	4.91 [1.33]	5.33 [1.48]	0.41 [0.60]	4.62 [1.48]	5.18 1.48]	0.56 [0.63]	*<.001*	.507	.290

ESW, MSW and LSW denotes early, mid and late swing, respectively. Min and Max denote minimum and maximum, respectively. Data are presented mean [SD] except for the total number of steps which is presented as the cumulative frequency of steps for each cohort and walking condition. One PD participant displayed 100% unimodal toe trajectories during preferred walking thus n = 35 for maximum toe clearance (early swing) and minimum toe clearance (mid-swing) and was excluded due to pairwise comparisons. Italicized values main and interaction effects p < .05.

### 3.2 Temporal-spatial gait determinants of MTC

**Fig. 2:**
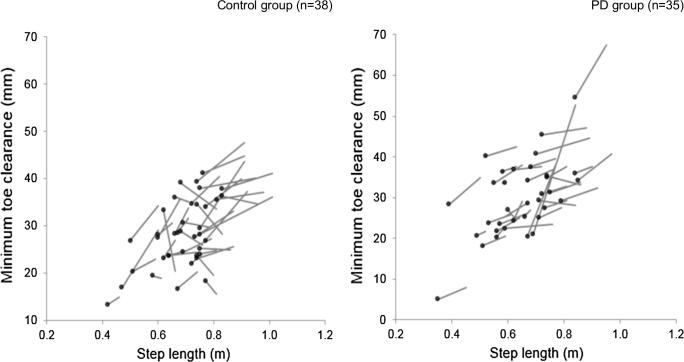
Vector plots demonstrating the change in minimum toe clearance with relation to the change in step length between preferred and fast gait velocity conditions in the control and PD groups. Data are presented for control (n = 38) and PD (n = 35) groups. Data is not included for one PD participant as they displayed 100% unimodal toe trajectories during the preferred gait velocity condition. The dot denotes the minimum toe clearance and step length obtained during the preferred gait velocity condition.

Correlations demonstrating the relationship between temporal-spatial gait and foot clearance for both groups and walking conditions are presented in the Supplementary Material. Linear regression models are presented in Table 2. Cross-sectional analyses revealed that step length and the walk ratio were consistently the strongest predictors of MTC for both groups and conditions (>28.3%). Vector plots revealed that all controls (n = 38,100%) and the majority of people with PD (n = 33, 92%) increased their step length from preferred to fast velocity (Fig. 2). Moreover, the majority of the control group (n = 27, 71%) and the PD group (n = 30, 83%) increased their MTC from preferred to fast velocity.

**Table 2:** Variance in the minimum toe clearance during mid-swing explained by each of the temporal-spatial parameters of gait for both groups and walking conditions.

		Preferred gait velocity				Fast gait velocity			
		R^2^ (%)	B	p	Rank	R^2^ (%)	B	p	Rank
Control	Gait velocity	13.3	12.962	.025	3	13.9	9.329	.021	4
n = 38	Swing velocity	12.1	10.675	.032	5	12.0	7.938	.033	5
	Step time	2.6	.024	.331	6	8.3	.045	.079	6
	Swing time	12.9	.075	.027	4	*17.7*	*.089*	*.008*	3
	Step length	*31.5*	*41.437*	*<.001*	2	*35.6*	*37.601*	*<.001*	2
	Walk ratio	*33.9*	*4005.956*	*<.001*	1	*40.0*	*4307.769*	*<.001*	1

PD	Gait velocity	18.2	14.988	.011	4	8.8	9.861	.079	4
n = 36	Swing velocity	12.5	11.563	.037	5	6.0	8.039	.150	6
	Step time	1.6	.022	.472	6	7.2	.049	.113	5
	Swing time	*21.9*	*.103*	*.005*	3	*22.8*	*.115*	*.003*	3
	Step length	*32.0*	*43.372*	*<.001*	2	*28.3*	*42.763*	*.001*	2
	Walk ratio	*34.7*	*4648.797*	*<.001*	1	*34.0*	*4634.574*	*<.001*	1

Temporal-spatial gait characteristics were ranked in order of the magnitude of variance in MTC explained. Italicized values denote significant contributors to regression models.

## 4 Discussion

This study is the first to explore the temporal-spatial mechanisms underpinning MTC in people with PD during overground walking. These novel findings suggest that step length is strongly related to MTC in both older adults and people with Parkinson’s disease and that those who walk faster, with longer steps, also lift their feet more.

### 4.1 Alterations in foot clearance during fast walking

Providing direct instructions to increase gait velocity may be considered an attentional strategy similar to being told to focus on taking “big steps”. As such the present results are in agreement with previous work which advocates the use of external (attentional) prompts to increase gait velocity and help to ameliorate the PD-associated differences in gait (Morris et al., 1994a, Morris et al., 1996). Previous work suggests that the neurodegeneration in motor control associated with PD gait evolves with greater changes in spatial scaling (step length and consequently gait velocity) compared to step time (Morris et al., 1996, Galna et al., 2015). When instructed to walk faster, people with PD tended to increase their step length more so than controls who reduced their swing time more so than people with PD (evidenced by changes in the walk ratio). In combination, these findings suggest a PD-associated difference in the temporal-spatial control of walking when increasing gait velocity. It is important to highlight that there is limited research documenting normative data for the walk ratio, particularly in PD populations, and as such it is unclear how clinically meaningful the group differences in the walk ratio are.

To date, there remains a lack of knowledge surrounding the pathogenesis of gait hypokinesia in PD (Morris et al., 1994b) and the associated alterations in temporal and spatial regulation of stepping patterns. Locomotor control is complex with multiple brain regions involved (Hamacher et al., 2015) and we understand that the basal ganglia and the supplementary motor area interact to regulate repetitive movements (Seitz and Roland, 1992). Further, it is this interaction that is thought to modulate the appropriate step length and cadence for a target gait velocity (Egerton et al., 2011). Considering the neurodegenerative nature of PD and the changes known to occur within the basal ganglia due to dopamine depletion, it is thought that the mechanism governing appropriate cadence and step length is damaged in people with PD. Evidence from cueing studies concurs that people with PD are able to achieve what may be considered a 'normal' step length (Morris et al., 1996). This suggests that the inability to internally initiate and regulate movement contributes to the changes in temporal and spatial control of walking in people with PD. Nevertheless, an increased gait velocity resulted in the same modifications in MTC in both groups. It is interesting to note that whilst controls walked quicker than participants with PD during both conditions, the group mean change in MTC (mid-swing) when increasing gait speed (from preferred to fast) was larger in the PD group. Previous work suggests that smaller alterations to step length in favour of increasing cadence when walking faster is associated with recurrent falls in older adults (Callisaya et al., 2012) and so interventions improving gait velocity through targeting step length may be advantageous for increasing MTC and minimising trip risk.

Our recent work indicated that foot clearance was altered in recently diagnosed participants with PD who present with a reduced peak toe clearance (late swing) and landing gradient compared to controls (Alcock et al., 2016). In the present study, the peak toe clearance (late swing) was the only significant group difference in foot clearance. This was initially surprising given the known gait impairments associated with PD. However, our previous study measured foot clearance during a 2-minute continuous walking protocol (compared to intermittent walks) when people with PD were walking more slowly (Alcock et al., 2016) and given the positive association between gait velocity and foot clearance (Cho et al., 2010, Alcock et al., 2016) the absence of PD-associated differences may be explained. Moreover, the majority of the group (89%) were classified as Hoehn and Yahr stage II and as such were considered to be of mild to moderate disease severity. Greater group differences are likely to emerge as disease symptoms advance.

### 4.2 Step length as a determinant of minimum foot clearance

A reduced magnitude of movement (hypokinesia) is associated with basal ganglia dysfunction in Parkinson’s disease, suggesting that defective cortical motor sets result in a miniaturisation of the gait cycle (Morris et al., 1994b, Morris et al., 1998). Our cross-sectional analyses revealed that step length and the walk ratio were the strongest contributors to explaining the variance in MTC in both groups. This finding has implications for the risk of falls in older adults generally (both with and without PD) and is in agreement with previous literature indicating that older adult fallers walk slower with shorter steps (Guimaraes and Isaacs, 1980) compared to older adult non-fallers who walk with an increased step length and reduced cadence regardless of walking velocity (Barak et al., 2006). Our data suggests that people who walk faster lift their feet higher and, more specifically, that people who walk with a longer step also have a higher MTC. Several studies have confirmed that training exercises can improve gait metrics in people with PD, particularly step length (Morris et al., 1994b, Herman et al., 2007, Herman et al., 2009, Bello et al., 2008) and based on the findings of the present study we would expect that increased step length would be accompanied by a higher MTC. However there have been no clinical trials evaluating whether adopting preventative strategies such as improving MTC may minimise trip-related falls risk and incidence. Given the potential for improving gait and foot clearance through therapeutic interventions, it presents as an attractive focus for falls prevention.

Our recent work in people with PD (Lord et al., 2016) concurs with the research literature in older adults (Abellan Van Kan et al., 2009, Verghese et al., 2009, Viccaro et al., 2011) that walking slowly is one of the strongest and most consistent predictors of falls. The results of the present study suggest that decreased gait velocity may, at least in part, be associated with reduced foot clearance which contributes to our understanding of the link between walking slowly and falls risk. Modifying the temporal-spatial control of walking in people with PD is possible through the implementation of cues (Nieuwboer et al., 2007) (i.e. visual, auditory, proprioceptive). Visual cues aim to reconfigure the spatial scaling of movement (Lewis et al., 2000) and as such they likely provide increased utility for increasing step length. Moreover, considering the ability to maintain or increase cadence in people with PD is retained, auditory cues may be more beneficial later in the disease course to regulate rhythmical stepping. Redirecting attention during gait through the use of direct instructions (e.g. to focus on walking performance or big steps) can result in significant improvements in gait velocity and step length in people with PD (Canning, 2005, Baker et al., 2007). Further work is required to confirm whether increased gait velocity and improved foot elevation may be achieved without compromising dynamic stability.

### 4.3 Study limitations

Measuring foot clearance during overground walking ensured that velocity was not constrained or manipulated (Lai et al., 2012) or kept constant (Sparrow et al., 2008) as is the case during treadmill walking. However, it must be considered that walking in the real world often presents heightened task demand due to navigating complex surfaces which may vary in height and material. Remote monitoring of foot clearance in the community is challenging given the need to model foot displacement relative to the floor surface. Despite this, the precise measurement of foot clearance is possible within controlled laboratory environments using a variety of methods including geometric modelling (Sparrow et al., 2008, Alcock et al., 2013) and segment digitisation (Startzell and Cavanagh, 1999, Loverro et al., 2013, Telonio et al., 2013). Measurement technique, analysis procedures and walking protocol utilised (i.e. overground *vs*. treadmill walking) all influence the variation in absolute MTC values reported in the literature. The measurement techniques used in the present study for quantifying foot clearance are less complex, however they permitted robust data collection for a large cohort in a longitudinal study without being burdensome. Furthermore, appropriate steps were taken to correct the signal for the measurement-induced offset resultant from variation in marker placement. This study assessed people with PD who were optimally medicated and wearing their comfortable shoes to preserve external validity. However, factors not controlled for which may have influenced foot clearance include visual function and correction (Johnson et al., 2007) and shoe sole geometry (Thies et al., 2015). Another factor influencing MTC is participant height, and whilst there were no statistical group differences in height, we re-ran the General Linear Model analyses controlling for height as a covariate. Each of the group differences were retained with the addition of only step length which just reached statistical significance (p = .042). Consequently, it was concluded that height did not have a significant influence on the findings presented and was not included within the present analyses. Lastly, research has proposed alternative MTC measures which may be linked with falls such as distribution and variability (Begg et al., 2007, Mills et al., 2008, Sparrow et al., 2008). These measures were not extracted in the present study due to an insufficient number of continuous steps collected over the four intermittent walks (a minimum of 200 steps (100 per foot) is recommended for calculating variability (Hamacher et al., 2014)).

Given the exploratory nature of this study we elected to not correct for multiple comparisons and this may be considered a limitation, however in the majority of cases the statistical differences we observed were considered highly significant (p < .002) and unlikely to be due to chance. Finally, whilst slower gait velocities have been successfully used to predict falls in people with PD (Lord et al., 2016), evidence confirms that this relationship is not linear and walking too quickly has also been linked to an increased risk of outdoor falls in particular (Quach et al., 2011). Falls are a multifaceted concern involving many influential factors often simultaneously and as such it is not advocated that increasing walking speed will reduce falls risk per se. However, in the context of PD symptoms including hypokinesia and bradykinesia, walking faster may reduce trip-risk due to improved foot clearance. Further complementary work is required to fully appreciate the mechanical underpinning of altered temporal-spatial gait and foot clearance in people with PD by quantifying lower limb joint kinetics and electromyography.

## 5 Conclusions

Attentional strategies offer the utility of increasing gait velocity and minimising pathology-associated gait deficits over short intermittent walks. This study has demonstrated that reduced MTC is associated with a smaller step in both older adults and people with PD. Interventions that target step length may result in increased MTC however their inclusion as a component of falls prevention requires further assessment.

## Conflict of interest

The authors have no real or perceived conflicts of interest to declare.
