# Supplementary material for: Step length determines minimum toe clearance in older adults and people with Parkinson’s disease
Source: J Biomech. 2018 Apr 11;71:30–6. doi: 10.1016/j.jbiomech.2017.12.002 (PMC5887869; doi:10.1016/j.jbiomech.2017.12.002)
Supplement: Supplementary data 1 [file mmc1.docx]

SUPPLEMENTARY MATERIAL 1 - Correlation matrix demonstrating the relationship between temporal-spatial gait parameters and foot clearance outcomes in both groups and walking conditions

|  |  | Preferred velocity | | | | | |  | Fast velocity | | | | | |
| --- | --- | --- | --- | --- | --- | --- | --- | --- | --- | --- | --- | --- | --- | --- |
|  |  |  |  |  |  |  |  |  |  |  |  |  |  |  |
|  |  | Gait velocity | Swing velocity | Length | Step Time | Swing time | Walk ratio |  | Gait velocity | Swing velocity | Length | Step Time | Swing time | Walk ratio |
| Older adults (n=38) |  |  |  |  |  |  |  |  |  |  |  |  |  |  |
| Max heel clearance (mm) | Preferred velocity | .603** | .595** | .862** | .160 | .426** | .848** | Fast velocity | .690** | .685** | .879** | .100 | .310 | .770** |
| Landing (heel) gradient |  | .422** | .366* | .615** | .115 | .386* | .616** |  | .559** | .503** | .740** | .067 | .325* | .662** |
| Max toe clearance (ESW) |  | .329* | .299 | .533** | .190 | .394* | .570** |  | .311 | .272 | .548** | .335* | .469** | .613** |
| Min toe clearance (MSW) |  | .364* | .348* | .561** | .162 | .359* | .583** |  | .373* | .347* | .596** | .288 | .421** | .632** |
| Max toe clearance (LSW) |  | .576** | .547** | .830** | .173 | .453** | .821** |  | .717** | .687** | .906** | .079 | .338* | .783** |
| Take-off (toe) gradient |  | .336* | .345* | .528** | .142 | .291 | .554** |  | .468** | .470** | .618** | .119 | .245 | .568** |
| Parkinson’s disease (n=36) |  |  |  |  |  |  |  |  |  |  |  |  |  |  |
| Max heel clearance (mm) | Preferred velocity | .666** | .642** | .854** | .056 | .478** | .845** | Fast velocity | .588** | .574** | .824** | .180 | .462** | .758** |
| Landing (heel) gradient |  | .060 | .080 | .071 | .028 | -.017 | .067 |  | .232 | .218 | .413* | .203 | .290 | .447** |
| Max toe clearance (ESW) |  | .438** | .367* | .578** | .119 | .467** | .598** |  | .323 | .272 | .551** | .250 | .470** | .588** |
| Min toe clearance (MSW) |  | .426* | .353* | .656** | .126 | .468** | .589** |  | .297 | .245 | .532** | .269 | .478** | .583** |
| Max toe clearance (LSW) |  | .633** | .596** | .767** | .045 | .432** | .731** |  | .607** | .598** | .786** | .102 | .393* | .680** |
| Take-off (toe) gradient |  | .264 | .207 | .406* | .141 | .410* | .458** |  | .134 | .077 | .458** | .414* | .582** | .607** |

ESW, MSW and LSW denote early swing, mid-swing and late swing, respectively. * p<.05, ** p<.01. Cells that are shaded in light (p<.05) and dark (p<.01) grey highlight significant correlations to aid visual interpretation
